# Towards an Active Role of Financial Institutions in Preventing Problem Gambling: A Proposed Conceptual Framework and Taxonomy of Financial Wellbeing Indicators

**DOI:** 10.1007/s10899-024-10312-8

**Published:** 2024-05-20

**Authors:** Nathan Lakew, Jakob Jonsson, Philip Lindner

**Affiliations:** 1 Centre for Psychiatry Research, Department of Clinical Neuroscience, Karolinska Institutet, Stockholm, Sweden; 2 Stockholm Health Care Services, Region Stockholm, Stockholm, Sweden

**Keywords:** Financial well-being, Financial institutions and gambling, Financial indicators, Financial harms, FWB measures

## Abstract

The transformation of gambling into a largely digital commodity has created a need for online payment technologies to facilitate online gambling, thereby also raising the question of what role these actors can play in the promotion of Responsible Gambling (RG). With the means and access they maintain, financial institutions are in a unique position to alleviate financial pitfalls, yet their role in the gambling context has thus far received little scrutiny. The objective of this study was to conduct an extant literature review to develop an initial set of financial indicators tailored for financial institutions, enabling them to engage in the RG initiatives. We conducted a two-step narrative literature review to identify both *general* Financial Well-Being (FWB) indicators across financial research disciplines, and one *specific* to gambling. A literature search over the past 20 years was performed across the following academic databases: Medline (Ovid), Sociological Abstracts (ProQuest), Web of Science (Clarivate), and PsycInfo (EBSCO). Manifest content analysis was used in step one to review general financial well-being, yielding a general FWB conceptual framework. In step two, we applied latent content analysis to the gambling-specific literature, linking essential concepts of gambling-related financial harms to the broader FWB literature. This resulted in a tentative taxonomy of indicators applicable to financial institutions with gambling customers. In tandem with the FWB conceptual framework, the preliminary taxonomy could provide a foundation for financial institutions catering to gambling customers to engage in the duty of care agenda, potentially broadening player protection beyond the current operator-focused RG measures.

## Introduction

Gambling disorder is unique among addictive disorders in that financial harm is a direct rather than indirect consequence, which often has a life-changing impact including psychological stress and even involvement in criminal activities (Langham et al., 2016; Li et al., 2017; Muggleton et al., 2021). Any large-scale gambling operation requires a financial institution to facilitate the transfer of money from (and to a lesser extent, to) customers. However, the role of financial institutions in preventing problem gambling (in addition to e.g. money laundering) has received little research attention (Swanton et al., 2019). This is striking, given the pace at which online gambling operators have integrated Financial technologies (Fintech) into the online gambling setting (Gainsbury & Blaszczynski, 2020; Nisbet, 2005). Fintech is now the preferred way to facilitate deposit and win withdrawals in online gambling, providing consumers with means to directly connect to their bank accounts – thereby enabling “access to uninterrupted funds” at one’s fingertips (Schüll, 2012).

The association between harmful gambling and financial difficulties is well established (Gainsbury, 2011; Muggleton et al., 2021; Swanton et al., 2019), however, for various reasons, there is a dearth of research on how financial institutions can potentially play a positive role in the financial state of their gambling customers. First, there seems to be a consensus in financial sectors that consumers are individually responsible for the outcome of their financial well-being (FWB) (Kempson et al., 2013). This mirrors the historical predominance of RG tools primarily targeting individuals or gambling operators (Adams, 2013; Harris & Griffiths, 2018). In addition, there is a lack of comprehensive framework outlining potential strategies for financial institutions to mitigate financial harm associated with gambling in their capacity as financial service providers (Swanton et al., 2019). The scarce research on conceptualizing how to onboard financial institutions to promote RG agenda is arguably a significant contributing factor that policymakers have historically been skeptical about mandating these institutions to play a positive role in the gambling context (Lakew, 2022).

Financial harms resulting from gambling activities share similarities with the financial difficulties one can experience that ultimately affect overall FWB. For instance, research shows that the first sign of gambling-related financial harm is the inability to fund “surplus purchases” such as holidays (Langham et al., 2016). Similarly, the loss of “financial freedom to enjoy life” is often cited in the general financial literature as the first manifestation of the FWB problems (Brüggen et al., 2017). Such overlaps suggest that the theoretical frameworks and models that are well-established in the financial research field can potentially provide a framework for how financial institutions can participate in promoting FWB among their gambling customers.

The purpose of the current study was to identify FWB concepts, frameworks, and indicators across the financial research domain and examine their potential relevance to studies on gambling-related financial harm. To achieve this objective, a narrative literature review was conducted in two stages. First, we performed a literature review encompassing discussions on general FWB across various research domains. This was followed by a review of FWB-related discussions within gambling field research to thematize existing financial indicators of gambling research within the broader FWB discourse. Drawing from the results, the study concludes by by examining the implications for policymaking, the discourse surrounding responsibility in fostering a healthy gambling environment, incentives for engagement in such initiatives, and delineating potential future research directions for financial institutions operating within the gambling context.

## Methods

This study employs a narrative review methodology. Narrative reviews are particularly useful when ‘one aims to link different research domains on the same topic, either for reinterpretation or interconnection’, and inspiration of new research ideas (Baumeister & Leary, 1997). Given that the present research aims to synthesize and reuse constructs within a specific knowledge area across different fields, the study design offers a suitable platform to accomplish our goal, namely conceptualizing a potential pathway for financial institutions serving gambling customers to engage in the RG agenda (Cronin et al., 2008). The narrative review was conducted in a two-step process. First, we identify the general concept of FWB and financial health indicators across myriads of research fields with two specific purposes: (1) to identify and categorize general FWB determinates; (2) to examine how these indicators and determinates are implemented to evaluate one’s financial health. The result of this analysis yielded a conceptual framework of FWB determinates and their indicators. In the second step, together with the conceptual framework, we investigated gambling-related financial harm literature using deductive-oriented latent content analysis to thematize FWB indicators into a gambling context. The second step results in a tentative taxonomy of FWB determinates alongside their indicators and manifestations in customer transactional data in the context of gambling activities.

### Search Strategy and Selection Criteria

A literature search was performed in the following databases: Medline (Ovid), Sociological Abstracts (ProQuest), Web of Science (Clarivate), and PsycInfo (EBSCO) with the last search conducted on 2022–11-22. The search strategy was developed initially on the Web of Science database, which was then translated into search terms for the other databases. No language restriction was applied. De-duplication was done using the method described by Bramer et al. (2016). One final, extra step was added to compare DOIs. The full search strategies and results of steps one and two for all databases are available in the appendix.

**Fig. 1:**
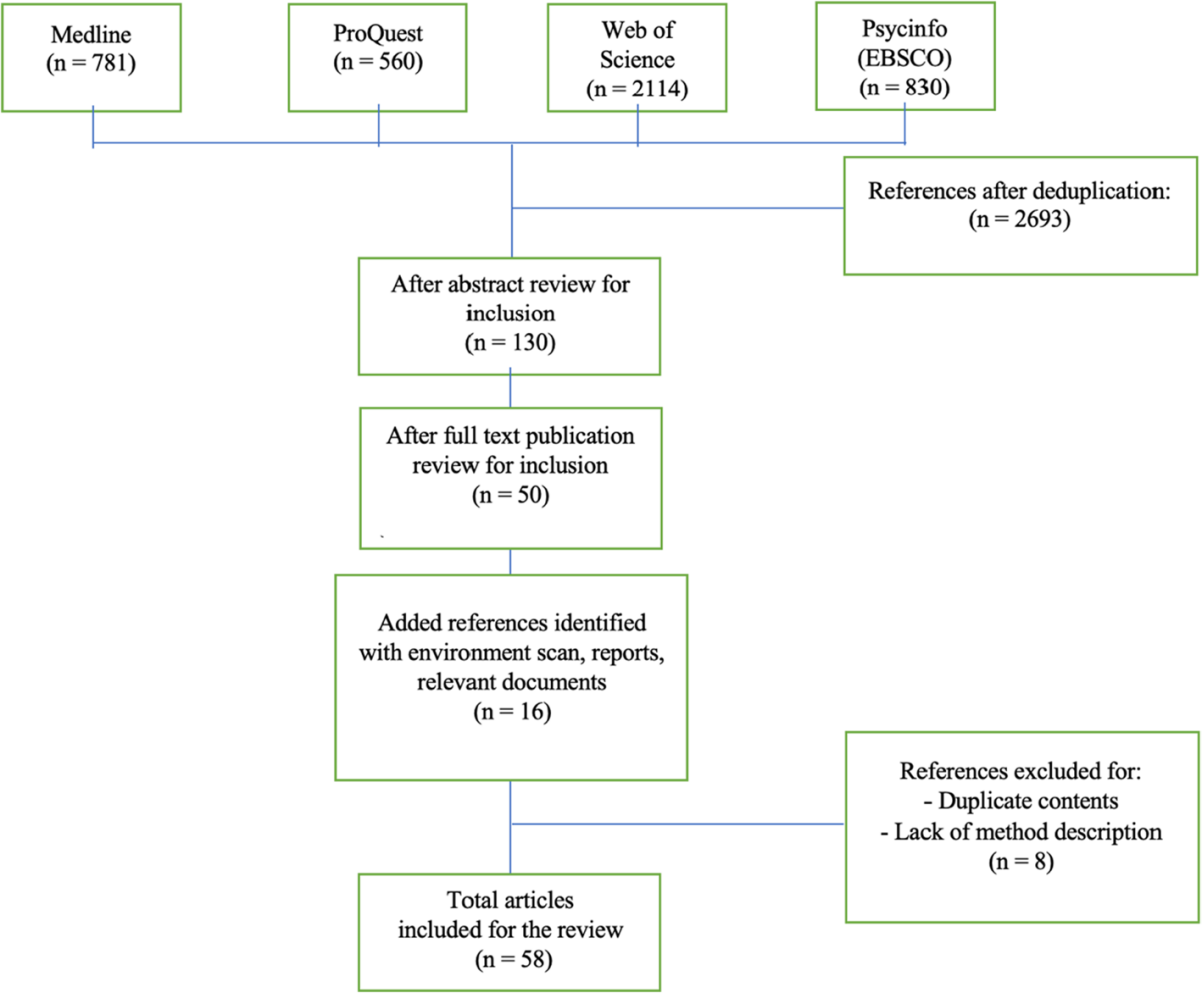
Step one literature selection process

For the first step of the general financial wellbeing review, the academic literature was searched using the following keywords: financial wellbeing; financ*; spending behavior; financial behavior; payment data behaviors; transaction data behavior; financial wellbeing capabilities. Articles relevant to FWB measurement indicators, manifestation of financial behaviors in transactional data, and components of FWB were selected for full-text review. Articles that were published more than 20 years ago, or had no available full text, were excluded from review. An environmental scan that focuses on financial institutions’ consumer financial health capability indicators was conducted to supplement the peer-reviewed journals. Figure 1 illustrates the literature selection process for step one.

**Fig. 2:**
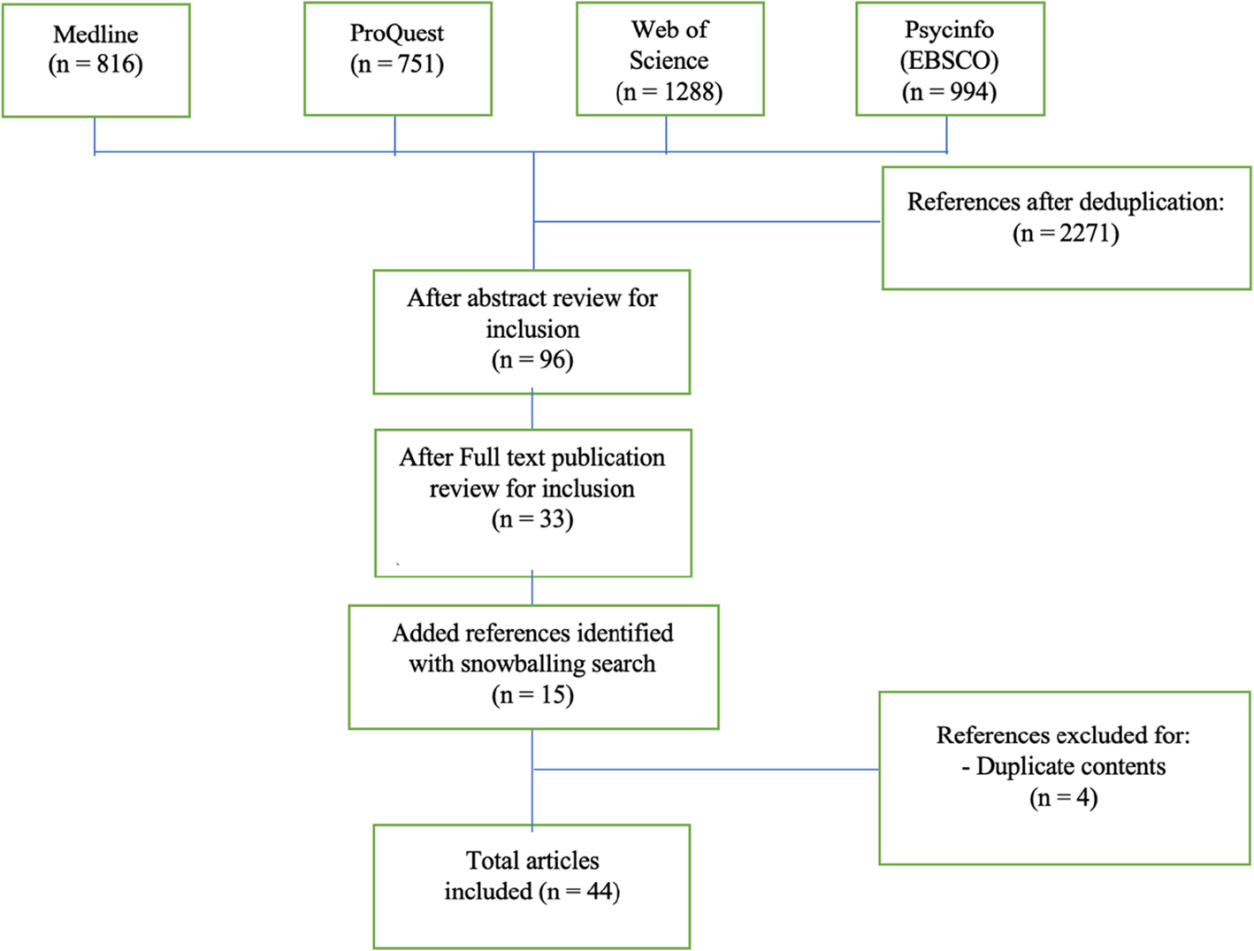
Step two literature selection process

In the second step of the review, manifestations of financial harms in the context of gambling were searched using the following keywords: gambl*; payment behaviors; financial harm; financial institutions; and financial data. Among other things, articles with a focus on gambling-related financial harms, financial transaction data in the gambling context, and the association of harmful gambling with deposits or withdrawal behavior were selected for latent content analysis. Based on the selected article, a snowball search method was then applied to complement the search result of literature that focuses on financial institutions within the gambling context. Articles for which full text was not available were excluded from the review (See Fig. 2).

### Synthesis of the Result

The literature review analysis involved both manifest and latent content analysis. Manifest content analysis was applied to the general financial wellbeing literature review result. Usually labeled as the ‘first order value of a data’, a manifest approach focuses on describing what is visibly observable in the data (Cash & Snider, 2014). The approach allows researchers to develop themes of ideas, or common features of studies, prior to deductive theory-based analysis. We employ the following steps in conducting the manifest content analysis. First, we imported all deduplicated references into Rayyan collaborative software for abstract review. After the abstract-based selection, the second step involved an inductive full-text analysis of articles. Each article was taken as a unit of analysis. Inductive categories were developed by first labeling each article based on their subject focus per se (e.g., transaction data analysis), and later by overarching theme (e.g., FWB determinates). Finally, associations between the themes enabled us to develop a FWB conceptual framework.

In the second step, a similar initial approach (i.e., abstract selection using Rayyan) was followed to select the final gambling context articles that were included for consideration. With the conceptual framework from step one at hand, the second step employed a deductive categorization scheme to start building themes. In doing so, a latent content analysis was used to infer financial behavior exhibited by players concerning the FWB determinates identified in step one. The latent analysis involves a deeper examination of the text to extrapolate underlying meanings and infer the relationship aspect of the subject under investigation (Graneheim & Lundman, 2004). Each article, as a unit of analysis, was examined to identify players’ gambling indicators that can be manifested in transactional data available to financial institutions serving gambling providers. These indicators were, in turn, latently considered through the lens of the FWB conceptual framework to create clusters of FWB determinates in the gambling context. In addition, non-transactional determinates and indicators identified in the broader FWB literature (e.g., socio-demographic factors) were sought in the gambling literature to determine the contextual outcome of FWB in the gambling context. Step two latent analysis involved multiple iterations of re-reading the selected articles and ‘discursive alignment of interpretations’ when different judgments of interpretation about the categorization of indicators occurred among the researchers (Seuring & Gold, 2012).

## Results and Discussion

### FWB in the Financial Literature

In the domain of financial research, it is evident that the concept of financial well-being can be formulated based on various factors Table 1. In their 25 years of bibliometric literature review of FWB, Singh and Malik (2022a) found that the discussion of FWB clustered around (1) the conceptualization and antecedents of FWB (2) the relationship between financial literacy and FWB, and (3) the consequences of FWB such as financial satisfaction. Furthermore, most of the studies were found to focus on identifying financial determinates and indicators associated with the FWB cluster(s) of the subject of these studies. The authors have additionally identified studies that focus on the development of metrics for determinates such as financial literacy or overall financial satisfaction.

**Table 1:** Examples of common concepts of and around FWB in the literature

Concept	Citations	Definition
Financial wellbeing:	Kempson et al.(2013)	“The extent to which someone can meet all their current commitments and needs comfortably and has the financial resilience to maintain this in the future.”
	Bruggen et al.(2017)	The perception of being able to sustain current and anticipated desired living standards and financial freedom
	Zaimah et al.(2013)	The subjective perceptions and objective indicators of individuals’ personal financial status
	Vosloo et al. (2014)	Objective and subjective aspects that contribute to a person's assessment of his/her current financial situation
	Consumer Financial Protection Bureau (2017)	“A state wherein a person can fully meet current and ongoing financial obligations, can feel secure in their financial future, and is able to make choices that allow enjoyment of life.”
	Shobha and Chakraborty (2017)	Yardstick for measuring individuals' financial security and their ability to make financial choices both in the present and future
Financial capability	Personal Finance Research Centre (2005)	“Positive financial behavioral connected with managing money, planning ahead, making the right choices, and getting help.”
Financial satisfaction	Joo and Grable (2004)	Contentment with one’s present financial situation with both the material (objective) and non-material (subjective)
Economic wellbeing	Sorgente and Lanz (2019)	Objective determinates of FWB such as material resources a person possesses

The work of Joo and Grable (2004), for example, aimed to develop financial satisfaction determinates, with demographic characteristics, financial stressors, individual behavior, and risk tolerance identified as determinates for financial satisfaction. Congruently, Kempson et al. (2013) identified key drivers of FWB as financial literacy, capability, as well as individual psychology and behavior. Moreover, their research on measuring FWB shows that both objective and subject matters tend to change over time. Further work on developing FWB measures and indexes can be found in the work of Prawitz et al. (2006) in which eight scaled questions were developed to evaluate both subjective feelings (i.e., confidence about future or financial resilience) and objective factors such as how often someone has situations where they ‘just get by’ in a given month. Another empirical work of scaled measurement is the Consumer Financial Protection Bureau (CFPB) set of ten questions, developed through cognitive interviews (CFPB, 2017). 

In most of the scaled measurements identified in the literature search, the concept of calculating a FWB score is used to create an individual’s financial wellbeing categories. For instance, the scoring used by the Australian and New Zealand governments has four FWB state categories: no worries, doing ok, getting by, and struggling – with each category being rated through a person’s score of FWB components: financial resilience, feeling financially comfortable, meeting everyday commitments, and feeling secure in the future (Kempson & Evans, 2022). We also found studies (Bruggen et al., 2017; Iannello et al., 2021; Prawitz et al., 2006) that focus on the manifestation of FWB determinants in everyday life. For example, spending habits, risky borrowing, or payday loans for daily consumption are used as indicators of financial behavior determinate which can be affected by external contexts such as consumer market, technology, or legal factors.

Finally, we found practice-oriented research with a focus on individuals’ financial behavior. In 2005, the UK financial regulator (2005) developed the notion of financial capabilities to improve one’s financial circumstance with a focus on behavior: ‘what consumers do/should do’ as opposed to ‘what they have/know’. These intervention-focused studies highlight skills such as money management, budget planning, financial product awareness, and access to financial assistance as the primary drivers of an individual’s FWB (Kempson et al., 2013).

**Fig.3:**
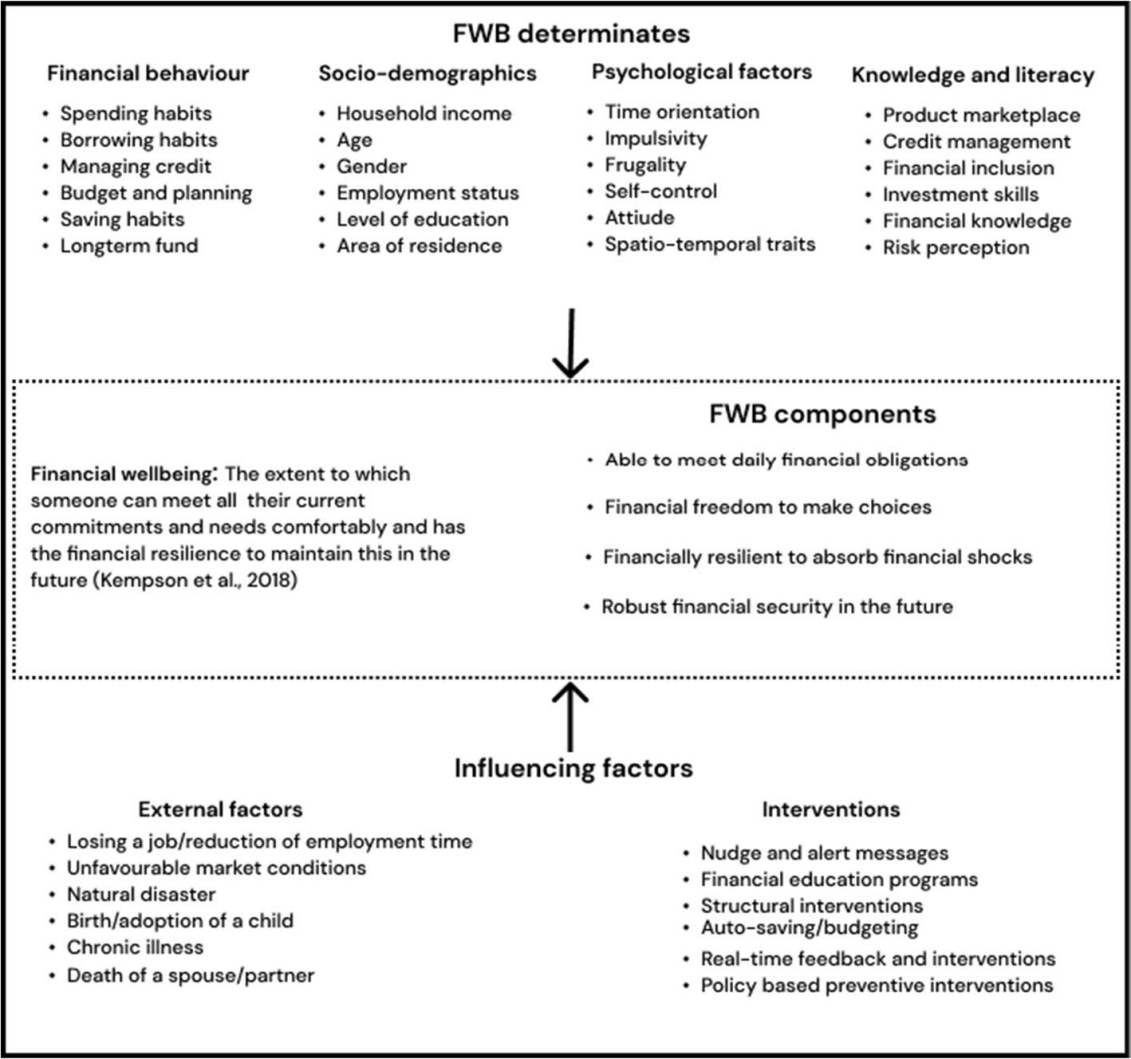
Conceptual framework of FWB determinates, their indicators, and influencing factors

In sum, research in the field shows that customers’ current and future financial resilience can be derived from key determinates such as level of financial literacy, financial behavior, and sociodemographic factors. In addition, indicators of these determinates, usually manifested in the form of behavior or financial decision-making, have been identified as indicators of future financial state. Figure 3 illustrates a FWB framework that encompasses these elements, including interventions, determinates and their indicators, external factors, and FWB components in the form of outcomes. In what follows, a summary of each element is presented.

### FWB Determinate and their Indicators

Four financial determinates were identified as containers for FWB indicators: behavioral, socio-demographics, psychological, and knowledge/literacy. Financial behavior consists of individuals’ actions in terms of managing their money which at times may also represent financial stress coping behaviors (Sorgente & Lanz, 2017). Financial behavior is a practice-focused determinate with tracking indicators spanning from an individual’s daily spending habits to long-term saving plans and credit management skills; hence financial behavior is at times used synonymously with financial capabilities. It is also where most of the intervention measures are focused. Other indicators associated with financial behavior include overspending, risky credit use, late bill payments, time orientation, current and future financial risk, and positive actions such as budget settings and retirement savings.

Socio-demographic determinates represent an individual’s social information (e.g., age, gender) and economic factors (e.g., household income). By one account, socio-demographics determinate accounts for 54% of an individual’s overall financial wellbeing outcome (Kempson & Evans, 2022). Parents’ social status has also been found to have a strong effect on young adults’ financial behavior (Xiao et al., 2011). Other indicators may include one’s own education level, country and area of residence, civil status, type of occupation, and status of employment. Psychology-based determinants include an individual’s cognitive abilities as well as personality traits and attitudes that can shape financial outcomes. In a large longitudinal study, Furnham and Cheng (2017) found that while childhood intelligence (at age 10) can predict future FWB among women, parent social standing in childhood has stronger associations with adult men’s FWB. In addition, personality-related features such as locus of control, low materialism, impulsivity, frugality, and self-esteem are routinely considered psychological determinate indicators of FWB (Brüggen et al., 2017; Hashmi et al., 2021; Iannello et al., 2021). Recently represented as financial knowledge and skill, financial literacy indicators include one’s ability in numeracy, digital literacy, knowledge of products, money management, and proactive actions such as budgeting (Bruine de Bruin & Slovic, 2021; Singh & Malik, 2022a). After behavior, financial literacy indicators are appropriate targets for proactive intervention efforts to improve individual FWB.

#### FWB Interventions

Two closely related determinates – financial behavior and literacy – are the common target areas of intervention. Financial literacy efforts are commonly developed to improve consumers’ understanding of their financial health including saving, budget planning, at times providing tools to do so. Intervention studies in financial behavior can be more proactive and target individual capabilities such as saving, spending, and investing through behavior change models including nudging, rewarding health behavior, or at times targeting structural changes through policies such as pension schemes (Brüggen et al., 2017; Kempson & Evans, 2022).

#### External Factors

At times included in the socio-demographic determinate, external factors constitute financial elements that affect an overall population such as inflation, economic development, political and legal factors, technological, and sociocultural factors, and personal life events such as job loss, divorce, and death in the family (Brüggen et al., 2017; Vlaev & Elliott, 2014). Though crisis management skills, access to help, and adaptions are seen as successful coping mechanisms, external factors can involuntarily shape an individual’s financial behavior.

#### Components of FWB

Finally, we found studies that focused on FWB components to measure an individual’s overall FWB and used to describe the consequences or legacy of determinates. Kempson et al. (2017), for example, identified four FWB components – meeting every day ends, feeling comfortable, resilience (ability to absorb financial shock), and security for the future – which they later used to categorize individuals into different wellbeing scales. Diener et al. (1999) found that psychological indicators notably affect the subjective component of FWB, and listed ‘health, family, leisure, and work’ as a critical component of FWB (Singh & Malik, 2022a). Brüggen et al. (2017) took a different approach where they delineated FWB components at different levels with different stakeholders – individual, collective, organizational, and societal levels. Table 2 summarizes our findings of FWB determinates, indicators, interventions, and components, uncovered in step one of the literature reviews. Note that the classification of these indicators is not an exhaustive list and that their effect on an individual’s FWB should be assumed to be the result of their collective and inter-acting influences.

**Table 2:** Financial determinates, indicators, and manifestations [Blair et al., 2022; Carlin et al., 2017; Furnham & Cheng, 2017; Kempson et al., 2013; Kempson & Evans, 2022; Singh et al., 2015; Singh & Malik, 2022b]

FWB determinates	Possible indicators	Examples of undesirable consequences	External factors	Common interventions
Financial behavior	• Spending habits • Borrowing habits • Managing credits and loans • Preference of credit over debit use • Rainy day emergency fund planning • General saving habits • Budget and keep track of money • Planning for future (pension fund)	• Unable to get credit/loan • Unable to meet ends • Missed payment • Borrowing to meet ends • No emergency fund • Late fees and reminders • Payday loan services • Over drafting • Unable to pay bills and on-going loans • Unable to meet commitments • Declining health • Financial and mental health stress • Anxiety • Unable to take financial shock • Violent behavior • Insecure future • Welfare reliance	• Losing a job/reduction of employment time • Legal and political factors • Unfavorable market factors • Technological factors • Natural disaster • Birth/adoption of a child • Divorce/separation • Chronic illness • Death of a spouse/partner	• Point of purchase interventions • Auto-save • Policy based preventive interventions • Auto-budgeting • Nudge and alert during irregular spending rhythm • Increasing level of spending friction • Financial education programs • Help sought to stabilizing vulnerable life situation • Structural interventions
Socio-demographics factors	• Household income • Age • Gender • Employment status • Civil status/partnership • State of residence (including mortgage) • Number of dependent children • Level of education (oneself and parents) • Provinces/area of residence			
Psychological factors	• Time orientation • Impulsivity • Locus of control • Frugality (deliberative thinking) • Self-control • Spatiotemporal traits (e.g., exploration) • Attitude to spending, money, and borrowing			
Financial knowledge and literacy factors	• Knowledge of product marketplace • Credit and interest management skills • Financial knowledge and risk perception • Health, household, and income Insurance • Financial inclusion • Investment and portfolio management skills			

### FWB in the Context of Gambling

#### Gambling and Consequences of Financial Harm

In most of the harmful gambling articles we reviewed, an individual’s FWB was identified as the first of the many problems players encountered and thereby considered the first sign of harmful gambling. Dimensions of financial harms have been developed based on their effect on individuals, families, communities, societal, or population levels (Abbott, 2020a, 2020b; Wardle et al., 2018). Furthermore, Langham et al. (2016) demonstrated the impact of financial harm by delineating it as a continuum, capable of manifesting as both an immediate crisis and a lasting legacy. Lack of FWB consequences identified in the literature includes trouble in meeting daily ends (e.g., reduced disposable income, not being able to cover basic needs or payday loans), lack of financial resilience (e.g., unable to take small financial shocks like buying a wedding gift), lack of future financial security (e.g., asset losses), and lesser financial choices (e.g., homelessness) (Hilbrecht et al., 2020, 2020; Langham et al., 2016; Latvala et al., 2022).

Harm has also been examined from a non-problem gambling perspective. Currie et al. (2017), for example, argued that non-problem gambling can still hold negative financial consequences affecting an individual’s ability to meet ends and financial resilience. Other studies, such as Farrell’s (2018), noted that harm to an individual’s general subjective well-being can be considered a wellbeing component effect of harm arising from gambling behavior.

#### Financial Determinates

The financial behavior determinates in the form of wagering and win withdrawal behaviors have been examined to detect early warning signs of harmful gambling. The literature identified and organized into this cluster tended to start with identifying determinate indicators (such as chasing losses) and work through identifying how such an indicator could be manifested in transactional data. For instance, Deng et al. (2019) identified indicators and their manifestations in transactional data in the form of gambling frequencies (e.g., active betting days), intensity (e.g., average bet size), or trajectory (e.g., variation of wager size over time). Congruently chasing behaviors can be detected by examining cumulative gains and losses across a given period where the deposit amount is consistently increasing (Ma et al., 2014).

Adami et al. (2013) observed that a betting pattern resembling a 'sawtooth'—characterized by escalating wager amounts followed by smaller bets (crashing bets)—indicates fund depletion. In addition, Haeusler (2016) showed that win withdrawal markers such as variance in amounts and reversing withdrawal patterns can also be used to detect harmful gambling. More financial behaviors such as the choices of payment method (e.g., credit vs. prepaid card use), late payments in the dunning process, small withdrawals accompanied by a steady increase in deposits, auto-deposit settings, and hitting limits are also associated with harmful gambling (Adami et al., 2013; Braverman & Shaffer, 2012; Haeusler, 2016). Psychological determinates such as impulsivity and self-control can also be manifested in transactional data. Catania and Griffiths (2022) noted that loss of control can be detected from an individual’s request to remove financial safety net tools such as budget settings. Smaller consecutive bets followed by a one-time arbitrary high bet are found to indicate an impulsive personality (Deng et al., 2019). In addition, the psychological state of being in a flow (Lavoie & Main, 2019), altitude, and motivation to win money in general, and the role of money in particular, have been identified to result in heightened gambling activities (Chen et al., 2012; Wulfert et al., 2008). While certain financial behaviors, such as wagered amounts may not be directly observable in the financial institution dataset, general patterns can potentially be inferred from deposit requests and winnings withdrawal, as these are closely linked to overall wagering behavior (Lakew, 2022).

Financial literacy in the form of understating the effect of specific gambling products on finances has also been a subject of interest. Markham et al. (2017) noted that some gambling products such as EGM are found to have a greater prevalence of financial harm. In one of the largest gambling transaction dataset studies we found, Muggleton et al. (2021) showed that financial literacy indicators such as one’s financial inclusion and knowledge about holding insurance, credit card use, or debt payment management skills, can be used as indicators of financial harm. In the same vein, Heiskanen (2017) noted that the conceptualization and meaning of money in a player’s life can determine their financial behaviors. Even though there exists ample research work on the effect of socio-demographic factors on gambling behaviors per se, we found few research that connects these factors with one’s financial wellbeing. Canale et al. (2017) examined gambling harms from the perspective of poverty and inequality. Low levels of education and neighborhoods with low social capital and household income have also been identified as risk factors for harmful gambling (Hilbrecht et al., 2020).

#### Interventions and External Factors

The discussion of external factors that affect one’s FWB, such as a breakdown in one’s social relationships, is customarily discussed under the umbrella of consequences of harmful gambling (Abbott, 2020a, 2020b; Langham et al., 2016). We have also found research that examines the increased use of digital technology and its effect on gambling behavior and financial health. Gainsbury and Blaszczynski (2020) noted that the proliferation of modern digital payment methods on gambling sites can result in easy depletion of funds through auto-deposit setting, direct redeposit of wins, or diminished value/lower awareness of spending. Cashless payment is generally found to affect lessening the so-called pain of paying; hence keeping individuals in the ‘state of flow’ and increasing ‘time on device’ – which both are associated with funds exhaustion (McGrath, 2006; Prelec & Loewenstein, 1998; Schüll, 2012). However, inconsistent results are reported whether the use of such payment solutions can exuberate harmful gambling (Palmer et al., 2021).

Similar to the general FWB research field, financial-oriented interventions in gambling research heavily focus on behavior determinate. Hurla et al., (2017) for example, suggested behavioral economics tools such as nudging and heuristics or ‘rule of thumb’ to facilitate beneficial behavior in decision-making. Technology-based interventions that include smart notifications of spending or real-time financial feedback are also proposed to avert harmful gambling (Lakew, 2022; Swanton et al., 2019; Wohl et al., 2017). In addition, intervention approaches in the gambling field tend to have a treatment orientation as opposed to prevention, though calls for policy and tech-tool-based preventive approaches are on the rise. Using the theory of affordance, Nower and Glynn (2022), for example, advocated for designing a regulatory policy that includes a robust assessment of what each player can afford before involving in gambling activities. Similarly, preemptive policies for non-problem gambling activities (Currie et al., 2009), default limits on digital payment accounts (Gainsbury & Blaszczynski, 2020), and an overall preventive public health approach have been proposed to curb harmful gambling (Livingstone & Rintoul, 2020).

### 3.4. Positioning Gambling within the Broader FWB Research and a Taxonomy of Indicators

The objective of this review was to explore a potential framework that financial institutions serving gambling customers could potentially adopt to advance the agenda of RG. By leveraging FWB as a lens, gambling-related financial determinates and their corresponding indicators can be established in a manner that resonates with financial institutions. Moreover, the general FWB intervention tools and approaches can offer insights into potential interventions that financial institutions can feasibly implement to support healthy spending behaviors in gambling. The present narrative review identified four overarching determinants of financial well-being: financial behaviors, socio-demographic factors, psychological elements, and financial literacy. In addition, external factors that affect one’s FWB, existing intervention approaches, and FWB components (outcomes) were identified.

Positioning the gambling financial literature within the larger FWB field, we found two prominent themes from our latent content analysis. The ‘outcome-focused’ studies – financial problems arise from harmful gambling – have to a larger degree influenced the extant research. To a lesser degree, and more recently, inferring harmful gambling based on financial behavior determinates has been examined based on financial data (Muggleton et al., 2021). We also found evidence that the role of money, monetary format, and financial institution’s responsibility and potential contribution to the effort of responsible gambling has been the subject of discussion (Heiskanen, 2017; Palmer et al., 2021; Swanton et al., 2019). The discussion of interventions, from both theoretical and practical level perspectives (e.g., public health approach or setting gambling budget limits), have also been examined to improve one’s subjective and financial wellbeing in the context of the gambling (Abbott, 2020a, 2020b; Farrell, 2018; Nower & Blaszczynski, 2010). Other general FWB determinates we identified – socio-demographic, external factors, and financial literacy – are either explored only in the context of gambling behavior (e.g., in the case of socio-demographics) or minimally acknowledged for their direct impact on players' FWB (e.g., in the case of financial literacy indicators).

The general FWB literature can provide a robust representation of financial indicators, their in-between interactions, key manifestations of these indicators, and intervention approaches that fit financial institutions’ framing of FWB. In line with such framing, Table 3 presents a catalog of FWB elements we identified in the gambling literature, organized in the form of taxonomy. The classifications presented in the taxonomy resemble the elements we identified in the FWB conceptual framework that can also be directly or indirectly observed from transactional data and/or in users’ profile information in financial institutions’ dataset. As such, the taxonomy is structured in the form of determinates, indicators, their manifestations in customer data, and possible interventions by financial institutions and policymakers. Personal data that financial institutions collect on their customers (e.g., a user contacts customer service because of a gambling problem) are included in the socio-demographic determinate as they are routinely considered as a user-profile related dataset.

**Table 3:** A taxonomy of FWB determinates, indicators, key manifestations, and intervention in gambling context [(Adami et al., 2013; Braverman & Shaffer, 2012; Catania & Griffiths, 2022; Chen et al., 2012; Deng et al., 2019; Gainsbury, 2011; Gainsbury & Blaszczynski, 2020; Gainsbury et al., 2018; Haeusler, 2016; Hurla et al., 2017; Livingstone & Rintoul, 2020; Muggleton et al., 2021; Nower & Glynn, 2022; Swanton & Gainsbury, 2020; Wohl et al., 2017; Xuan & Shaffer, 2009)]

FWB determinates	Possible indicators	Key manifestations	Possible interventions
User profile and socio-demographic factors	• Profile dataset • Provinces/area of residence • Socio-economic group • Household characteristics • Country • Level of education • Player’s interaction with Customers Service (CS)	• Customer profile information 1. Age 2. Gender 3. Income information 4. Multiple phone/emails associated with account 5. Education • Unfitness marking in credit score • Previously blocked account • Being rude to customer service • Contact CS for gambling problems • National public registry/credit service • Blaming gambling loss on financial services • Asking for a bonus	• Financial institutions 1. Financial screening to evaluate individuals’ ability to gamble 2. Account based tailored real-time feedback about spending intensity 3. Visualize feedback on gambling expenditure reports compare with others; including wins and loses 4. Switching behavior notifications to break ‘flow’ in auto-deposit settings 5. Score based categorization of state of problem gambling 6. Limit of deposit or auto-deposit settings 7. Financial literacy lessons in form of heuristics on gambling spending 8. Customer care calls • Intervention policies 1. Enforce gambling affordability testing 2. Legislatives to ensure that payment gateways have FWB responsibilities to their gambling customers as much as operators and players 3. Prohibition of credit gambling or ensure safeguards implementation
Financial behavior	• Deposit behavior • Debt payment history • Payment methods • Win withdrawal behavior • Credit gambling behavior	• Number of debt reminder (e.g., SMS, emails) • Multiple payment methods used to make a deposit • Making consecutive deposit request of equal amount to the same gambling operator (auto-deposit setting) • Sudden drop in wager after a steady wager increase^*^ • Irregularly decreasing wager amount in a session^*^ • Fluctuation of deposit toward lower trajectory • Increase in the number of frequencies in gambling financial transactions • Rejection of deposit by gambling operator • Steady increase in wager accompanied by small win withdrawals^*^ • Rejection of deposit by financial institution (e.g., card issuing company) • Merchant rejection of win withdrawals • Card registration attempt refused • Account subjected to bailiffs • Getting lower deposit amount than requested	
Psychological factors	• Attitude to money • Impulsivity • Time orientation • Locus of control • Frugality (e.g., limit behavior)	• Continuous deposit to same site • a one-time arbitrary high deposit • Betting spree^*^ • Wagering all wins at once^*^ • Increasing wager size in a short period of time^*^ • Arbitrary increase in wager after a steady bet amount^*^ • Cancelling withdrawals • Frequently hitting a budget limit • Requesting for raising budget ceiling • Making wager on multiple gambling sites in short period of time^*^	
Financial knowledge and literacy factors	• Product knowledge (e.g., gambling segment choices) • Use of FWB tools • Money management skills • Proactive action on spending • Payment method preference	• Merchant category code (MCC) numbers • Requests for setting betting limit • Similar wager ceiling in each period (e.g., monthly) • Using pre-paid cards instead of direct bank account methods • Continues to wager despite unpaid credit and multiple reminders • Asking for credit payment pause	

^*^These key manifestations can be inferred via* deposit and/or withdrawal behavior patterns*

The classification of indicators can be used to target specific consumer FWB weaknesses and devise personalized interventions (e.g., impulsive spending with strict betting limit). Moreover, indicators we found in determinates such as socio-demographics can help to develop general preventive policy measures that, for example, set a limit on how much an individual can afford to gamble within a specific period. Finally, rooted in financial concepts, the taxonomy can serve as a base to advance: (1) available indicators for financial institutions to assess harmful gambling behaviors, (2) the potential use of individuals' FWB as a perspective to gauge if a customer can engage in gambling activities, thereby enabling preventive measures, and (3) potential policy measures, responsibilities, and incentives for financial institutions serving gambling customers to engage in RG efforts.

## 4. Scope and Limitation

The current research has a limited focus aimed at exploring feasible frameworks (i.e., FWB) to onboard financial institutions into the RG agenda. However, implementing such an initiative may entail practical, political, and ethical considerations that are beyond the scope of this research. For instance, there exists a strongly polarized debate in the field on how gambling research should conceptualize the issues of responsibility in gambling activity (Blaszczynski et al., 2022). On the one hand where gambling harms are perceived as a subjective experience resulting from the intensity of the gambling activities (i.e., dose), emphasis is given to individual responsibility to mitigate gambling risks (Shaffer et al., 2020). On the other hand, we can find the theoretical framing of gambling harms as a preventable public health disorder with a focus on promoting wider cross-sectoral responsibilities applicable at a population level (Johnstone & Regan, 2020). Moreover, 'external factors' such as individuals' financial circumstances, psychological as well as cognitive biases, and behaviors might contribute to risky spending habits. While existing literature underscores the adverse effects of problem gambling on financial health (Muggleton et al., 2021; Swanton et al., 2019), establishing a clear causal relationship between FWB and problem gambling can pose challenges. These might raise questions regarding the extent to which financial institutions should bear accountability for the FWB of their gambling customers.

Closely related to this challenge is the difficulty in rallying support for a cross-sectoral RG agenda where the ‘trade-off’ is, at times, politically portrayed as limiting individuals’ freedom to choose a recreational activity (Korn et al., 2003). Recent research has advocated for a middle ground where all stakeholders in gambling, including financial institutions, are urged to embrace responsibility in adopting RG initiatives (Gainsbury et al., 2020). However, formulating suitable incentives for all participants in gambling to promote active involvement in RG can present a challenge. Furthermore, there is a general lack of effective industry models concerning ethics and responsibility in the gambling context, and current structures such as corporate social responsibility (CSR) are perceived as overtly favorable to business interests to serve as effective platforms for RG engagement (Tetrevova, 2023).

There have been recent developments mandating financial institutions to undertake duties traditionally assumed by gambling operators – including treating operator customers as their customers – particularly if their substantial revenue comes from serving gambling customers (Finansinspektionens, 2022). Recent legislation in Sweden concerning third-party participants such as Fintech which serves gambling operators has now explicitly prohibited from serving offshore gambling operators where some Fintech has already faced penalties for non-compliance with these regulations (Spelinspektionen, 2023). As such, current trends suggest that policy-based incentives for financial institutions with gambling customers are on the rise, however, whether such a ‘responsibility-sharing’ trend would extend in the future to include harms from gambling activities remains to be seen.

Finally, we want to stress that items in the presented taxonomy are subjective, and defining individuals’ FWB state is best done through a concerted interaction between the indicators. As such, the present catalog of determinates and indicators would be of better use if seen as more of a starting point to facilitate the development of robust measurements. When finalizing the taxonomy, we went through key manifestation items to ensure that all items were captured by the corresponding indicator items. With methods such as cognitive interviews or quantitative survey questionaries, it is possible to empirically test key manifestation items, and consequently create scales to categorize an individual’s FWB state in a gambling context (Swanton et al., 2021).

## 5. Conclusion and Future Research Agenda

With the interdisciplinary nature of the gambling industry's growth, it can be argued that the responsible gambling agenda should not solely focus on gambling operators and players. The present study investigated the potential role, and direction for, payment providers serving gambling operators to engage in responsible gambling initiatives. Given that players tend to use one payment provider across multiple gambling sites, RG measures implemented at the provider level can have a wider positive effect on consumers’ gambling spending. In addition, payment institutions have a unique position to overview players’ cross-operator behavior of gambling – hence can offer a better evaluation and detection opportunities of harmful gambling. However, a body of literature suggests that ‘payment institutions with gambling customers lack comprehensive policies and strategies’ to engage in RG efforts (Hurla et al., 2017; Swanton et al., 2019). Our extant literature review illustrates that players’ financial aspects can be conceptualized through the lens of general FWB research, which can help to integrate financial institutions in responsible gambling initiatives.

**Table 4:** Research agenda for financial institutions with gambling customers

1. The role of online payment institutions in gambling: Fintech companies are increasingly becoming the primary facilitators of deposits and withdrawals in online gambling. It is imperative to formally conceptualize their role within the context of gambling. We recommend: • Examining the pitfalls of using Fintech method in gambling context • Identifying directions and frameworks to onboard financial institutions to the effort of RG • Devising policies and regulation that fits with Fintech’s (1) effect on gambling behavior, and (2) rapid adoption across gambling operators
2. FWB determinates: Financial determinates can be used to detect, intervene, and prevent gambling harms. We recommend to: • Further contextualize and validate FWB determinates both theoretically and empirically • Explore how external and cultural factors collectively affects financial determinates and indicators • Identify how and which determinates affect which population groups including across types of gambling games and socio-demographics
3. Developing interventions: With Fintech account-based solution, it is now possible to create, for example, personal FWB scorecard for gambling activities. We recommend to: • Further examine how to develop a scale based FWB score that can be used to categorize players in to, for example, gambling-health categories based on their financial state • Identify which financial state/categories can be influenced by which determinates, and determinate based interventions (e.g., impulsive deposit behavior to stringent limit intervention) • Explore the possibilities of preventive policies that includes verifiable and nationwide assessment of players regarding their ability to afford gambling
4. Collaboration between policies, academics, operators, and Fintech: As gambling activities became interdisciplinary, it is imperative to bring all actors to the table. We suggest for: • Policymakers to carefully (re)examine financial license criteria to include gambling harm minimizations tools in cases where the institution in question has gambling customers/substantial revenue from gambling • Fintech with gambling customers to include routine screening for harmful spending habits, and provide provisions such as budget limiting tools for their players • Researchers to collaborate with Fintech industry to further develop and empirically test FWB models • Gambling operators who decide to integrate Fintech for their payment handling need to validate if the Fintech in question is in line with their responsible gambling policies
5. Responsible practices, incentives, and interventions: Further research is warranted to examine what constitute as corporate ethical responsibilities and potential incentives associated with third-party actors in gambling context. We suggest to: • Explore potential models and forms of responsibility to integrate financial institutions into the RG agenda (i.e., extending CSR) • Aligned with their service offerings, examine conditions and incentives for enablers companies with indirect role in gambling activities to motivate participation in RG efforts (e.g., policies, expansion of regulatory rules) • Examine duty of care and RG possibilities covering third party actors such as payment solutions providers to offset potential harms from increasing adoption of digital payment solutions

The research area of financial institutions in the gambling context is relatively new. Drawing from the findings, the current study highlights potential research directions for the field to involve financial institutions in the RG agenda, as summarized in Table 4. First, as Fintech companies have increasingly become the preferred way of handling deposit and win withdrawals in online gambling, their influence on gambling behavior and potential direction to onboard them to the RG agenda need formal conceptualizations. Second, as Fintech enables the gathering of account-based payment history, there exists an opportunity to provide a personalized intervention, including prior evaluation of individuals’ FWB, real-time feedback based on spending behavior as well as develop FWB score model to evaluate and categorize the level of financial state among players. Furthermore, the integration of gambling with other research fields such as finance, corporate responsibilities, and technology studies emphasizes the importance of interdisciplinary research and collaboration across sectors involving researchers, industry stakeholders, and policymakers.

Finally, the fast adoption of online gambling has attracted profit-driven third-party actors such as Fintech to the field who lack RG knowledge nor are currently mandated by regulations to participate in gambling duty of care measures. Their growing involvement has led to uncharted territory regarding the responsibilities they should undertake, if any, and the types of incentives that can motivate their engagement in such duties. (e.g., policies and regulations). Consequently, the ongoing ‘political’ discourse around responsibilities, obligations, and incentives in gambling activities should start incorporating third-party gambling actors, such as financial institutions.

## Appendix I – Topic: General financial wellbeing

### Documentation of search strategies

Date: November 2022.

*Databases*:

1. Medline (Ovid)
2. Sociological abstracts (ProQuest)
3. Web of Science (Clarivate)
4. Psycinfo (EBSCO)

*Total number of hits*:

- Before deduplication: 4,285
- After deduplication: 2,693

## Appendix II – Topic: Gambling and financial wellbeing

### Documentation of search strategies.

Date: November 2022.

*Databases:*

1. Medline (Ovid)
2. Sociological abstracts (ProQuest)
3. Web of Science (Clarivate)
4. Psycinfo (EBSCO)

*Total number of hits:*

- Before deduplication: 3,849
- After deduplication: 2,271
